# Data-driven modelling of mutational hotspots and in silico predictors in hypertrophic cardiomyopathy

**DOI:** 10.1136/jmedgenet-2020-106922

**Published:** 2020-07-30

**Authors:** Adam Waring, Andrew Harper, Silvia Salatino, Christopher Kramer, Stefan Neubauer, Kate Thomson, Hugh Watkins, Martin Farrall

**Affiliations:** 1 Wellcome Centre for Human Genetics, University of Oxford, Oxford, UK; 2 Department of Medicine, University of Virginia, Charlottesville, Virginia, USA; 3 Radcliffe Department of Medicine, University of Oxford, Oxford, UK; 4 Oxford Medical Genetics Laboratories, Churchill Hospital, Oxford, UK

**Keywords:** cardiomyopathy, clinical genetics, genetics

## Abstract

**Background:**

Although rare missense variants in Mendelian disease genes often cluster in specific regions of proteins, it is unclear how to consider this when evaluating the pathogenicity of a gene or variant. Here we introduce methods for gene association and variant interpretation that use this powerful signal.

**Methods:**

We present statistical methods to detect missense variant clustering (*BIN-test*) combined with burden information (*ClusterBurden*). We introduce a flexible generalised additive modelling (GAM) framework to identify mutational hotspots using burden and clustering information (*hotspot* model) and supplemented by in silico predictors (*hotspot+* model). The methods were applied to synthetic data and a case–control dataset, comprising 5338 hypertrophic cardiomyopathy patients and 125 748 population reference samples over 34 putative cardiomyopathy genes.

**Results:**

In simulations, the *BIN-test* was almost twice as powerful as the Anderson-Darling or Kolmogorov-Smirnov tests; *ClusterBurden* was computationally faster and more powerful than alternative position-informed methods. For 6/8 sarcomeric genes with strong clustering, *Clusterburden* showed enhanced power over burden-alone, equivalent to increasing the sample size by 50%. *Hotspot+* models that combine burden, clustering and in silico predictors outperform generic pathogenicity predictors and effectively integrate ACMG criteria PM1 and PP3 to yield strong or moderate evidence of pathogenicity for 31.8% of examined variants of uncertain significance.

**Conclusion:**

GAMs represent a unified statistical modelling framework to combine burden, clustering and functional information. *Hotspot* models can refine maps of regional burden and *hotspot+* models can be powerful predictors of variant pathogenicity. The *BIN-test* is a fast powerful approach to detect missense variant clustering that when combined with burden information (*ClusterBurden*) may enhance disease-gene discovery.

## Introduction

The clustering of pathogenic missense variants in specific regions or domains of proteins has been frequently reported.^1–5^ A plausible mechanism underpinning this phenomenon is the presence of multiple loss or gain-of-function variants within functionally important domains.^6^ Despite numerous examples of variant clustering, there have been few attempts to explicitly model variant residue position as a predictor of pathogenicity.^7^

Mendelian disease genes were historically identified by linkage and candidate gene studies in multiplex affected families.^8^ With technical advances in high-throughput, exome sequencing has become another approach to identify novel pathogenic genes and variants. The aggregated burden of rare variants in affected cases compared with healthy controls has proved to be a useful test to confirm candidate^9^ and identify novel,^10^ putative pathogenic genes. Several enhancements to this simple approach have been developed including weighting by variant frequency or functional annotation,^11^ integrating additional genetic risk factors such as polygenic risk scores^12^ or modelling both protective and deleterious variants by comparing variance in variant-level case–control frequencies.^13 14^ However, due to sample size limitations, few methods exist to test the rare disease ultra-rare variant hypothesis in a case–control setting. Furthermore, there are no compelling examples where rare variants play a protective role. Here, we detect association based on a dominant model of rare deleterious variants and demonstrate that power can be increased by including variant residue position alongside gene-level burden. Unlike previous approaches to address this problem,^7 15^ we present computationally fast methods, for a realistic Mendelian disease genetic model, that place equal weight on the burden and clustering signals, making it a viable alternative strategy where simple burden testing has been unsuccessful.

The American College of Medical Genetics and Genomics (ACMG) has produced guidelines to interpret variant pathogenicity.^16^ These guidelines integrate diverse data and classify variants into five categories from benign to pathogenic. However, due to limited information available for many variants, they fall into the category ‘variant of uncertain significance’ (VUS). Although positional information is covered by criteria PM1 (‘Located in a mutational hot spot and/or critical and well-established functional domain (eg, active site of an enzyme) without benign variation’), there is a lack of robust statistical evidence for mutational hotspots, resulting in inconsistent application of this criterion. Furthermore, although much work has gone into the development of in silico prediction scores, alternative scores can be conflicting, leading to discordance among testing laboratories^17^ and uncertainty in their application (criteria PP3: ‘Multiple lines of computational evidence support a deleterious effect on the gene or gene product’). However, wherever large patient cohorts are attainable, mutational hotspots and the uncertainty surrounding in silico predictors can be directly estimated from the data.

Hypertrophic cardiomyopathy (HCM), a relatively common autosomal dominant disease (1 in 500 prevalence), is a major cause of heart disease in people of all ages^18^ and a cause of sudden cardiac death. In our cohort, eight sarcomeric genes collectively provide firm molecular diagnoses for ~27% of HCM patients, with a further ~13% of patients carrying a VUS in the same genes. It has been suggested that disease and gene-specific approaches are needed to improve interpretation,^19^ and guidelines have been produced for specific genes and/or disease areas.^20–23^ HCM is common enough to provide the large datasets needed for these gene-specific and data-driven approaches.

Here we propose new statistical approaches to explicitly include variant residue position in rare missense variant association and interpretation: *BIN-test* for detecting clusters of rare missense variants and *ClusterBurden* to combine this with burden information for association testing and generalised additive models (*GAMs*) for hotspot estimation and modelling of in silico pathogenicity prediction algorithms. We apply these methods to a large cohort of 5338 HCM patients and up to 125 748 GnomAD^24^ population controls. We demonstrate that using positional information increases power to detect disease–gene associations and elucidate the clustering signals present in 34 cardiomyopathy genes. We then use *GAMs* to model mutational hotspots and pathogenicity predictors for six core sarcomeric genes.

## Methods

### Patient cohorts and simulated data

Next-generation sequence data for 34 cardiomyopathy genes (online supplementary table S1) were available from two large HCM cohorts (online supplementary method S1A); 2757 probands referred to the Oxford Medical Genetics Laboratory (OMGL) for genetic testing and 2636 probands recruited to the Hypertrophic Cardiomyopathy Registry (HCMR) project.^25^ Additional genome-wide SNP array data permitted exclusion of closely related individuals (ie, ≤3rd degree) for the HCMR cohort using the KING relationship inference software,^26^ and comparable data were unavailable to reliably identify closely related OMGL samples. High-coverage exonic sequences were captured by target enrichment and sequenced on the MiSeq platform (Illumina Inc). Joint bioinformatic processing of both datasets followed the Genome Analysis ToolKit version 4 best practice guidelines (online supplementary method S1B). OMGL variants were confirmed by Sanger sequencing, and HCMR variants were manually checked by inspection of BAM files.

10.1136/jmedgenet-2020-106922.supp1 Supplementary data

10.1136/jmedgenet-2020-106922.supp2 Supplementary data

The GnomAD exomes population reference database was used as a control group, which includes variant frequency data based on up to 125 748 individuals. For both cases and controls, only missense variants with a GnomAD population maximum allele frequency of less than 0.0001^9 10^ were included. This excludes potentially common ancestry-specific variants that are unlikely to be pathogenic for HCM.

### Detecting missense variant burden and clustering: *ClusterBurden*

Current methods to discover novel Mendelian disease genes focus on the burden of rare variants in an affected cohort relative to controls. Here we develop a powerful approach to detect differences in rare missense variant positions between two cohorts named *BIN-test*. We propose an approach that combines burden information (Fisher’s exact test) with clustering signals (*BIN-test*) into a single framework: *ClusterBurden*. This framework tests the joint hypothesis of an excess of rare missense variants *and* differential clustering in case–control data. This was accomplished by combining the p values from a burden test with the *BIN-test* using Fisher’s method.^27^ As there are no known examples of a protective burden of rare exonic variants in cardiomyopathy, we only consider an excess burden in the case group, making it a one-sided test. An important assumption of this method is that the contributing p values are independent; this was assessed in simulated data by Spearman’s rank correlation test.^28^

As the background distribution of variant residue positions may be non-uniform, a cluster of variants observed in affected cases is insufficient to determine association with disease. Therefore, to detect disease-relevant clustering, distributions were compared between affected cases and unaffected controls. We propose *BIN-test* to evaluate these distributional differences. First, the protein’s linear sequence of amino acid residues is split into *k* bins of equal length for each cohort. A χ^2^ two-sample test is applied to the resultant *k* × 2 contingency table of binned variant counts. The null hypothesis is that the relative frequency of observed variants in each bin is the same for cases and controls. Significance depends on how many bins deviate from this expectation and by how much. We applied a *k* ~ *n* ^2/5^ heuristic^29^ to select the optimal number of bins (*k*) dependent on *n*, the total number of observed variants. We compared the performance of the *BIN-test* with two other tests that compare distributions between two samples: Anderson-Darling (AD)^30^ and Kolmogorov-Smirnov (KS).^31^ Power and type 1 error were calculated using the (*r+1)/(n+1*) estimator where *r* represents the number of simulated datasets with p values less than 0.05 and *n* is the number of simulations.^32^ To adjust for uneven sequencing coverage between the cohorts, the aggregated counts in each *BIN-test* bin were adjusted by the reciprocal of the mean 10× coverage across that bin for each cohort. For the burden test, sample sizes were similarly adjusted by the mean 10× coverage over the entire gene (online supplementary method S2).

To determine the theoretical performance of *ClusterBurden*, synthetic data were generated using a forward-time simulator (online supplementary method S3), designed to imitate rare variants in genes with discrete exonic regions of increased pathogenic potential. Six different scenarios were considered: three clustering scenarios (uniform, a single pathogenic cluster and multiple pathogenic clusters) and two protein lengths (500 and 1000 amino acid residues). For each scenario, 10 000 synthetic datasets were generated with 5000 cases and 125 000 controls. Variants were filtered by their frequency in simulated controls at a minimum allele frequency (MAF) of <0.0001. The performance of *ClusterBurden* was compared with two published position-informed rare variant association tests (RVATs), DoEstRare^7^ and CLUSTER,^15^ and three position-uninformed RVATs: C-alpha,^14^ SKAT^13^ and WST^33^ (online supplementary table S2). Subsequently, Fisher’s exact test, *BIN-test* and *ClusterBurden* were applied to our HCM-GnomAD case–control data across the 34 cardiomyopathy gene panels. Post hoc analytic power calculations were performed by treating *Clusterburden* and Fisher’s exact test as likelihood ratio tests, with non-centrality parameters scaling with sample size.^34 35^

### Hotspot estimation and in silico predictor modelling using GAMs

To test the hypothesis that a variant’s position can improve pathogenicity interpretation, we considered gene-specific models of variant clustering in cases and controls. By combining information on gene-level burden and variant positions, these data-driven models estimate the regional burden across the linear protein sequence to quantify mutational hotspots. The models were fitted in the GAM framework,^36^ implemented in the R package ‘mgcv’.^37^ The outcome variable was disease status, so each model was unsupervised with respect to previous classifications of pathogenicity. The training data included all rare missense variants in cases and controls, including known pathogenic variants in the control set. Therefore, this approach implicitly models incomplete penetrance and benign background variation, leading to unbiased estimates of variant ORs.

GAMs, as an extension of the linear modelling framework, are designed to deal with non-linear relationships of unknown complexity, between explanatory variables (eg, residue position) and the response variable (eg, case–control status). When a relationship is potentially non-linear, it is represented by a smooth curve instead of a straight line. These curves are inferred automatically using restricted maximum likelihood, which reduces overfitting by penalising excessive ‘wiggliness’.

Using this framework, we defined the structure of the *hotspot* model, which models carrier status (gene-level burden) and residue position (clustering). To incorporate gene-level burden, non-carriers must also be modelled. However, as variant-level features such as residue position are meaningless for non-carriers, a nested model structure is required, whereby residue position is included *only* as an interaction with carrier status. Under these circumstances, the smoothed residue position term is multiplied by zero for non-carriers, excluding this undefined data from the model. The structure of the *hotspot* model is as follows:

*P* = β_0_ + β_1_ *carrier_status* + s_1_ (*residue_position,* by=*carrier_status*) + **ε**

where *P* is the probability of being a case, β_0_ is the model intercept, β_1_ is a linear coefficient for *carrier_status*, s_1_ is a smoothed (ie, non-linear) function for *residue_position*, by is used to generate factor–smooth interactions and ε is a binomial error term. To account for uneven sequencing coverage between the cases and controls, the contribution of each datum to the log-likelihood was weighted by the reciprocal of the mean 10× coverage in the surrounding region (online supplementary method S2).

The feasibility of this approach is dependent on the number of observations, thereby limiting its application in our data to the six core sarcomeric genes: *MYBPC3*, *MYH7*, *MYL2*, *MYL3*, *TNNI3* and *TNNT2,* each carrying at least 20 rare missense variants. A *hotspot* model was produced for each gene, and raw model predictions for each residue position, in the form of logistic probabilities and SEs, were transformed to ORs and 95% CI. There is currently no universal guidance on how to quantitatively apply ACMG criteria PM1. However, using the probability that a variant is a case variant as a proxy of pathogenicity, we can use predicted probabilities to attribute levels of evidence. Here we stratify variants based on the probability thresholds 0.9, 0.95 and 0.99 to represent supporting, moderate and strong evidence of pathogenicity. These correspond to ORs of approximately 10, 20 and 100.

As GAMs are additive in structure, it is straightforward to include further predictors in the model. Here we experimented with the inclusion of variant prediction scores extracted from the dbNSFP4.0 database for nonsynonymous SNPs' functional predictions.^38^ These in silico prediction algorithms are covered by the ACMG criteria PP3, however, like criteria PM1, it is challenging to apply this criterion quantitatively. It is unclear which threshold determines a pathogenic variant with a given probability and whether these thresholds are consistent across genes. Both of these problems can be solved using gene-specific models, as the relationship between in silico predictors and disease status is inferred. Furthermore, uncertainty on the usage of these scores can be quantified.

To avoid overfitting, a strict two-stage feature selection procedure was implemented. In stage 1, dbNSFP features (online supplementary figure S3A) with a marginal p value<0.002 were selected (0.05/24 Bonferroni corrected). In stage 2, backwards elimination was implemented, whereby features with the lowest p value are removed one at a time until all features are significant (Bonferroni corrected for the number of features selected in stage 1). The resulting models (online supplementary figure S3B), which are henceforth termed *hotspot+* models, assimilate evidence of gene burden, variant clustering and pathogenicity prediction scores.

10.1136/jmedgenet-2020-106922.supp3 Supplementary data

Model performance for these GAMs are best judged by the estimates of uncertainty accompanying predictions. However, to determine the relative ability of these models to predict pathogenicity, they were compared with models based on single in silico predictors and expert variant classifications. Relative performance was assessed using the receiver operator characteristic area under the curve (AUC) across cross-fold validations performed by dividing the data into 10 random training and test sets using an 80%:20% ratio. Hotspots were also compared with those identified by Walsh *et al*,^39^ based on a partially overlapping dataset, using a one-dimensional clustering algorithm (henceforth abbreviated as 1dC) and constrained-coding regions (CCR) identified by Havrilla *et al*.^40^

## Results

### Testing the hypothesis of clustered missense variants

Under the null hypothesis of no excess burden or clustering, the false-positive rate of the *BIN-test* and AD test were adequately controlled in simulated data, whereas the KS test was overly conservative (online supplementary table S3). The *BIN-test* had superior power than AD or KS under all clustering scenarios and protein lengths with, on average, 1.8-fold more power to detect clustering. As power covaries with the number of observed variants, power was higher for longer proteins as well as proteins with larger pathogenic regions.

Correlations between p values generated by the *BIN-test* and Fisher’s exact test were compared for simulated data under: (1) a null model of no association or (2) a disease model of overburdened and clustered variants. For the disease model, there was a positive correlation (Spearman’s rank correlation rho=0.40) between p values, as anticipated as the power of these tests covaries with the number of observed variants. However, under the null model, the p values were completely uncorrelated (rho=0.00, p=0.5), satisfying the independence assumption of Fisher’s method.

The false-positive rate for *ClusterBurden*, DoEstRare, CLUSTER and C-alpha were all well controlled in the simulated datasets (online supplementary table S3). On the contrary, SKAT and WST showed markedly inflated false-positive rates under the null and were not examined further. *ClusterBurden* was the most powerful method when clustering was present with an average of 72% power, 3% higher than the second-best test DoEstRare. The best method under the uniform model (ie, burden-only) was CLUSTER, which had ~5% more power than *ClusterBurden*. Among the position-informed RVATs, *ClusterBurden* was the most rapid to compute taking less than a second per gene, whereas DoEstRare and CLUSTER took over 20 or 4 min, respectively.

**Figure 1:**
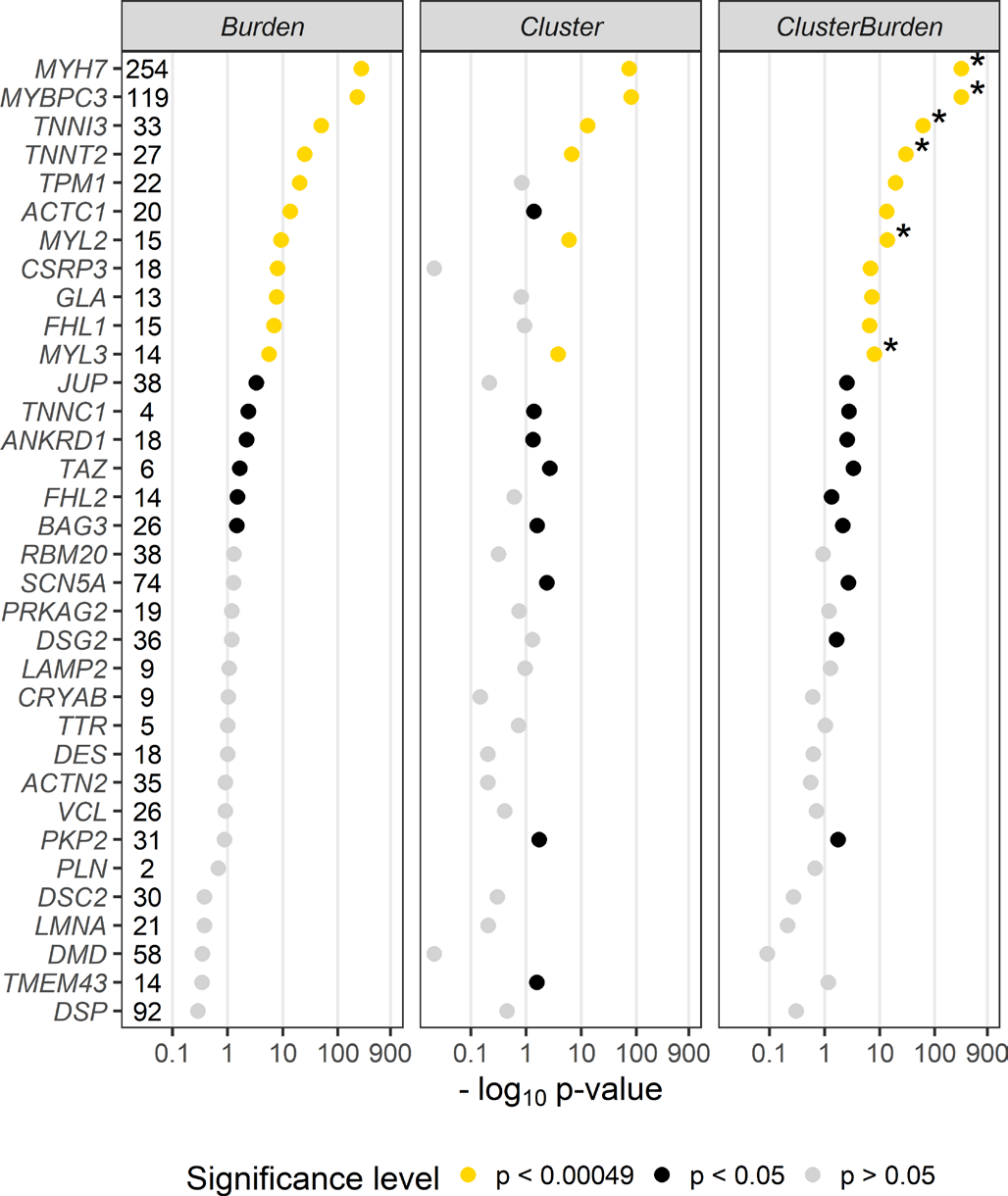
P values (−log_10_) from a Fisher’s exact test (*Burden*), *BIN*-test (*Cluster*) and *ClusterBurden* across a 34-gene cardiomyopathy gene panel. Our case–control dataset contains 5338 hypertrophic cardiomyopathy cases and 125 748 GnomAD controls. For all tests, only missense variants with a *popmax* MAF less than 0.01% were considered. The number of observed case variants in each gene is displayed next to the gene symbol. P values displayed in yellow are significant after Bonferroni correction for 34 genes × three tests (p<0.00049), p values in black are nominally significant (p<0.05) and p values in grey are insignificant (p>0.05). Asterisks denote genes where the *ClusterBurden* p value is lower than the burden p value.

We then examined 34 cardiomyopathy genes for rare missense variant associations with Fisher’s exact test (burden), *BIN-test* (cluster) and *ClusterBurden* (combined cluster and burden) in our cohorts of HCM cases and GnomAD controls (figure 1). Significance thresholds were conservatively Bonferroni adjusted to allow for 34 genes × three methods (ie, p values adjusted for 102 tests to p<0.00049). Significant burden signals were then detected in 11 genes with Fisher’s exact test; *MYH7* (p<5.84 × 10^−265^), *MYBPC3* (p<1.43 × 10^−222^), *TNNI3* (p<1.96 × 10^−50^), *TNNT2* (p<1.08 × 10^−25^), *TPM1* (p<5.79 × 10^−21^), *ACTC1* (2.81 × 10^−14^), *MYL2* (4.89 × 10^−10^), *CSRP3* (9.29 × 10^−9^), *GLA* (1.77 × 10^−8^), *FLH1* (1.32 × 10^−7^) and *MYL3* (2.64 × 10^−6^). The *BIN-test* detected significant clustering for six core sarcomeric genes: *MYH7* (p<1.50 × 10^−74^), *MYBPC3* (p<1.19 × 10^−81^), *TNNI3* (p<9.28 × 10^−14^), *TNNT2* (p<2.16 × 10^−7^), *MYL2* (p<1.08 × 10^−6^), and *MYL3* (p<1.7 × 10^−4^). Two additional sarcomeric genes showed nominal evidence of clustering; *ACTC1* (p<0.0412) and *TPM1* (p<0.0494). *ClusterBurden* confirmed the association for 11 genes that showed burden signals and calculated substantially lower p values for the six core-sarcomeric genes with significant clustering. Post hoc power calculations demonstrate the empirical enhanced power for this approach in true disease-causing genes. For example, for *MYL2* a 53% increase and for *MYL3* a 52% increase in sample size would be required for the burden-alone test to have equivalent power to *ClusterBurden*.

### *Hotspot* and *hotspot+* models

**Figure 2:**
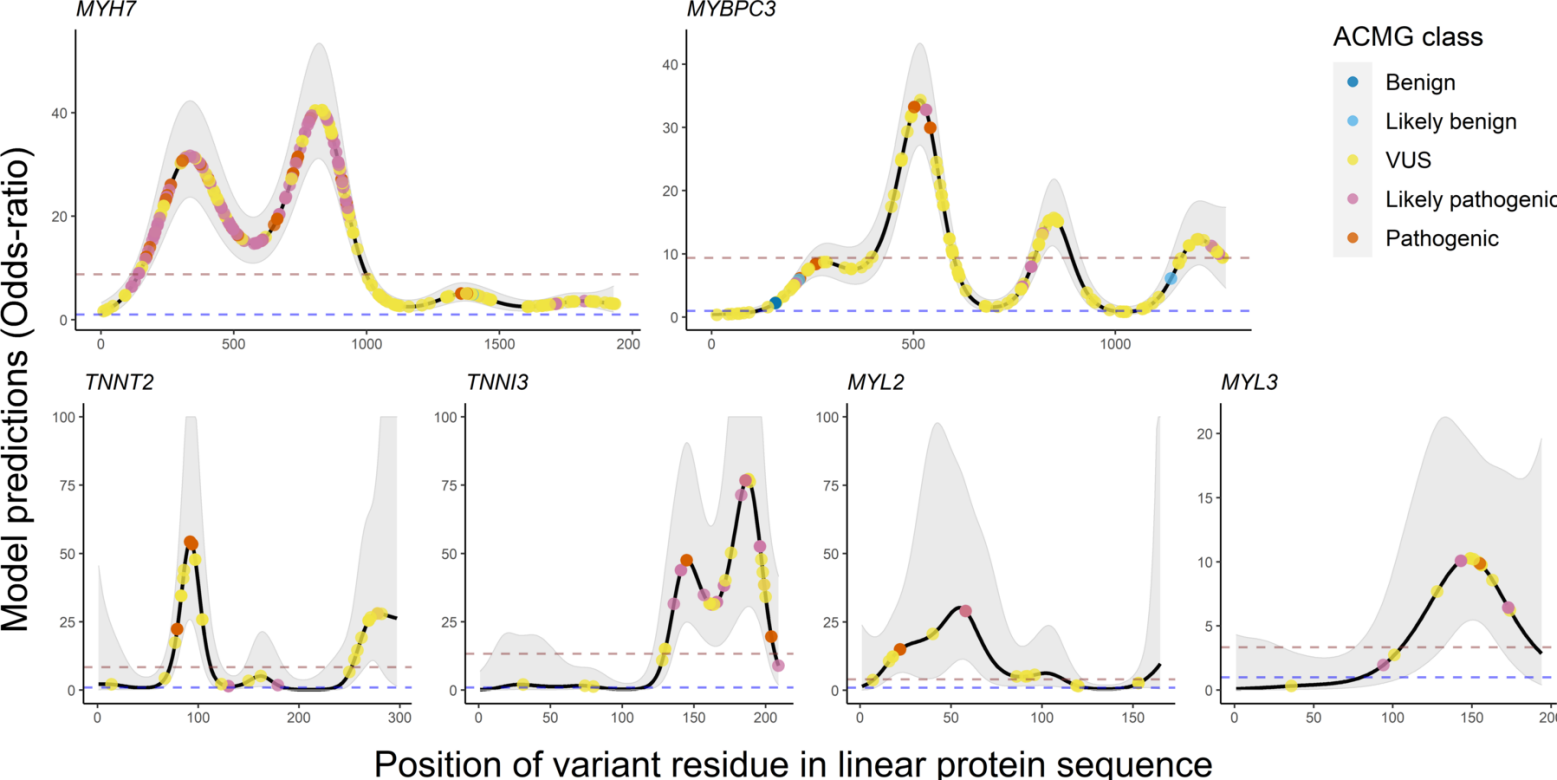
OR predictions and 95% CIs for *hotspot* models. Mutational hotspots were modelled for six firmly established HCM disease genes. The 95% CIs for model predictions are displayed as light grey shading. A dashed blue line at OR=1 indicates the threshold at which a region is in excess in cases or >1 or depleted in cases or <1. Rare missense variants in the HCM data are superimposed on the predicted model and coloured by their ACMG class assessed by Oxford Medical Genetics Laboratory. ACMG, American College of Medical Genetics and Genomics; HCM, hypertrophic cardiomyopathy; VUS, variant of uncertain significance.

Figure 2 summarises the *GAM* predictions for six sarcomeric genes in the *hotspot* models. Visualising the predicted ORs for each residue illuminates the local burden of rare missense variants across each protein, identifying ‘mutational-hotspots’ and highlighting areas of potential functional importance in HCM pathogenesis. Confidence in these predictions are tight for *MYH7* and *MYBPC3. C*onversely, the genes with fewer observed variants have much broader CIs. ORs from all models correlate strongly with expert manually assigned classifications, though there is substantial overlap between classes. Variants with a VUS classification show the highest spread in predictions (online supplementary figure S1).

**Figure 3:**
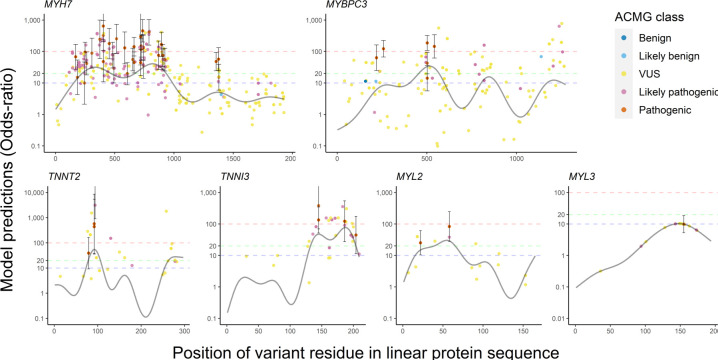
OR predictions and 95% CIs for *hotspot+* models. Points denote rare missense variants in the HCM dataset and are coloured by their ACMG classification assessed by Oxford Medical Genetics Laboratory. ORs on the y-axis are displayed on a log_10_ scale and were derived from the *hotspot+* models; incorporating gene burden, residue position and gene-specific significant secondary features from dbNSFP. The solid black curvy lines represent the predictions for each residue in the protein for a gene burden and position model (*hotspot* model). Regions above the blue (OR=10), green (OR=20) and red (OR=100) dashed lines ascribe supporting, moderate and strong evidence, respectively, for the combined PM1 and PP3 criteria. ACMG, American College of Medical Genetics and Genomics; HCM, hypertrophic cardiomyopathy; VUS, variant of uncertain significance.

Figure 3 displays *hotspot+ model* predictions (modelling burden, position and in silico predictors) for individual variants in the same six genes. Due to strict feature selection, the number of predictors included in each model depends on the power to detect associations between features and disease status. This resulted in fewer features for genes with fewer observed variants; *MYL3* had no additional significant features. As residue position is included as a predictor in each model, predictions generally follow the *hotspot* model; however, due to additional in silico predictors, ORs tend to vary from this pattern, stratifying risk for variants at the same position.

As with the *hotspot* models, there was a strong correspondence between predictions and expert classifications (mean rho 0.41 across six models). In *MYH7*, mean predicted ORs for pathogenic, likely pathogenic and VUS variants observed in cases, were 74, 50 and 20, respectively. Again, the VUS class had the highest heterogeneity, with predicted ORs ranging from 0.25 to 197 (*MYH7*). Half of these VUSs are observed in a single case and absent in controls (private singletons). The empirical ORs for these variants, based on the case and control frequencies and adding 0.5 to zero-count cells (Haldane continuity correction^41^), had wide 95% CIs: 44.9 (1.5 to 1338.3). However, *predicted* ORs for such variants could have greater precision with different point estimates depending on the precise amino acid substitution. In *MYH7*, five of these singleton VUSs had predicted ORs greater than 100 and three had ORs less than 1.

**Figure 4:**
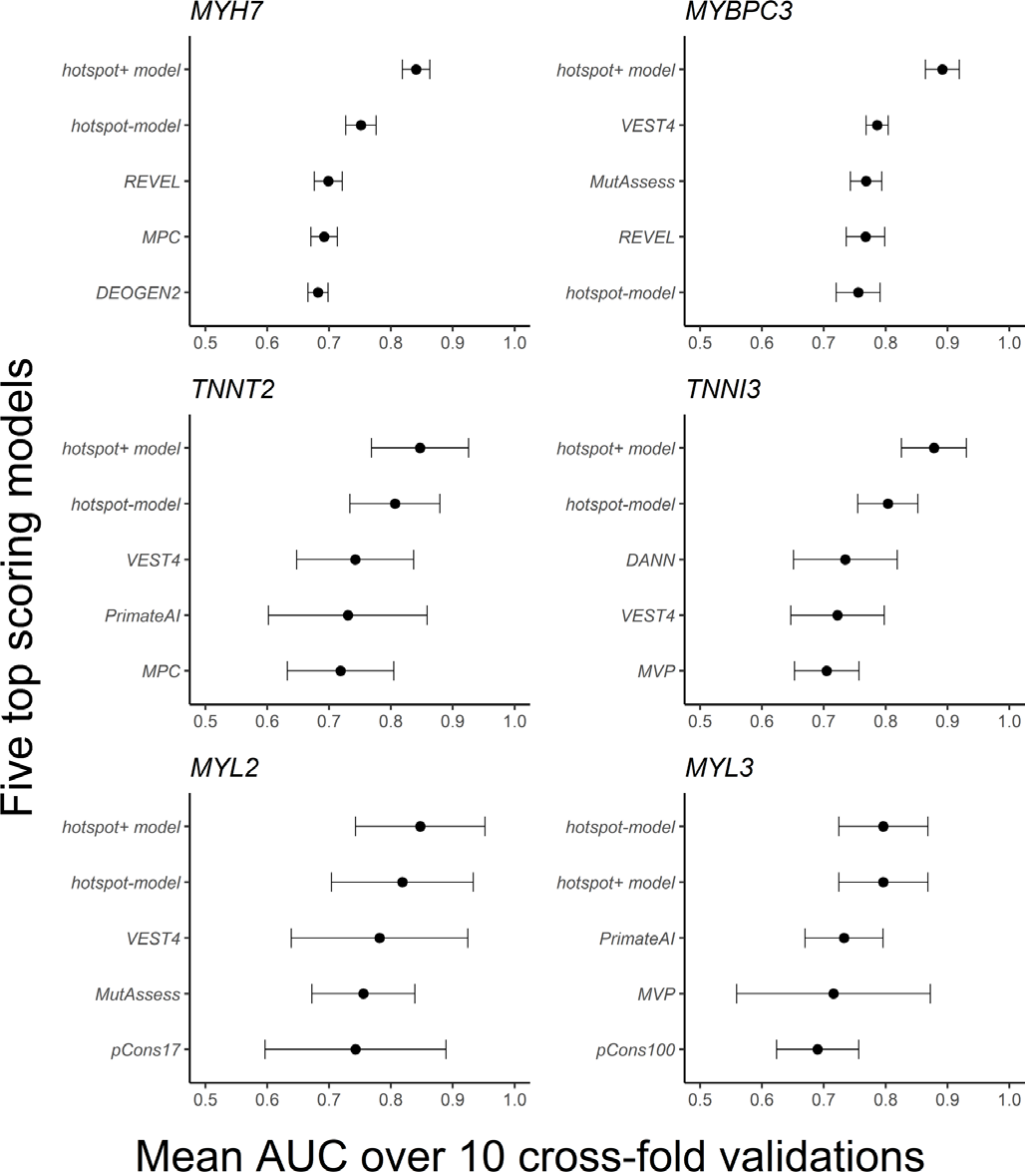
Means and SD of area under the curve (AUC) across 10 crossfold validations. For each gene, the *hotspot+* model and *hotspot* model are compared with each individual in silico predictor from dbNSFP. Each model is trained on the same HCM-GnomAD case–control missense variants all filtered at a GnomAD population maximum frequency of 0.01%. Only the five highest mean AUC scoring models are displayed (for full data see online supplementary figure S2). HCM, hypertrophic cardiomyopathy.

The mean and SD of AUC for 10 crossfold validations summarise overall model performance (figure 4; online supplementary figure S2). The *hotspot+* model had a much higher mean AUC than any individual in silico predictor in isolation. With the exception of *MYBPC3*, the *hotspot* model outperformed any standalone score from dbNSFP. This suggests that residue position is more important in determining pathogenicity than the in silico predictors in dbNSFP for these sarcomeric proteins. The AUC SD for *MYH7* and *MYBPC3* were the smallest, suggesting they have the highest capacity to generalise to new variants.

The GAMs were then used to attribute evidence of pathogenicity based on the ACMG criteria PM1 and PP3. Using the ACMG OR thresholds described in the methods, table 1 shows the proportion of variants in our cohort with evidence of pathogenicity predicted by the *hotspot* and *hotspot+* models. For the *hotspot* model, the PM1 criteria was satisfied, with supporting (OR >10) or moderate (OR >20) evidence, for some variants in all genes except *MYL3.* Strong evidence of pathogenicity (OR >100) was not predicted by the *hotspot* model for any variant. Conversely, the *hotspot+* model, which combined criteria PM1 and PP3, provided strong evidence of pathogenicity for many variants, including VUSs in *MYH7*, *MYBPC3*, *TNNT2* and *TNNI3*.

**Table 1:** Proportion of variants with evidence of pathogenicity in *hotspot* and *hotspot+* models

	*VUS*				*Likely pathogenic*				*Pathogenic*			
	*N*	*Sup. (%)*	*Mod. (%)*	*Str. (%)*	*N*	*Sup. (%)*	*Mod. (%)*	*Str. (%)*	*N*	*Sup. (%)*	*Mod. (%)*	*Str. (%)*
*Hotspot* models												
*MYH7*	***123***	10	28	0	***93***	33	55	0	***36***	31	61	0
*MYBPC3*	***97***	35	12	0	***13***	38	23	0	***6***	33	67	0
*TNNT2*	***19***	21	47	0	***4***	0	50	0	***4***	0	100	0
*TNNI3*	***18***	17	67	0	***11***	9	91	0	***4***	0	100	0
*MYL2*	***12***	25	0	0	***1***	100	0	0	***2***	100	0	0
*MYL3*	***10***	0	0	0	***3***	0	0	0	***1***	0	0	0
*Hotspot+* models												
*MYH7*	***123***	18	26	4	***93***	16	58	14	***36***	8	75	17
*MYBPC3*	***97***	16	25	7	***13***	15	54	15	***6***	17	33	50
*TNNT2*	***19***	16	26	16	***4***	0	50	25	***4***	0	50	50
*TNNI3*	***18***	22	33	22	***11***	18	45	36	***4***	0	50	50
*MYL2*	***12***	0	25	0	***1***	0	100	0	***2***	50	50	0
*MYL3*	***10***	0	0	0	***3***	0	0	0	***1***	0	0	0

Each observed variant across six genes in our HCM cases was given supporting (OR >10; Sup.), moderate (OR >20; Mod.) or strong (OR >100; Str.) evidence of pathogenicity based on model predictions from the *hotspot* and *hotspot+* models. The proportion of variants with supporting, moderate or strong evidence are stratified by expert classifications made by Oxford Medical Genetics Laboratory.

HCM, hypertrophic cardiomyopathy.

**Figure 5:**
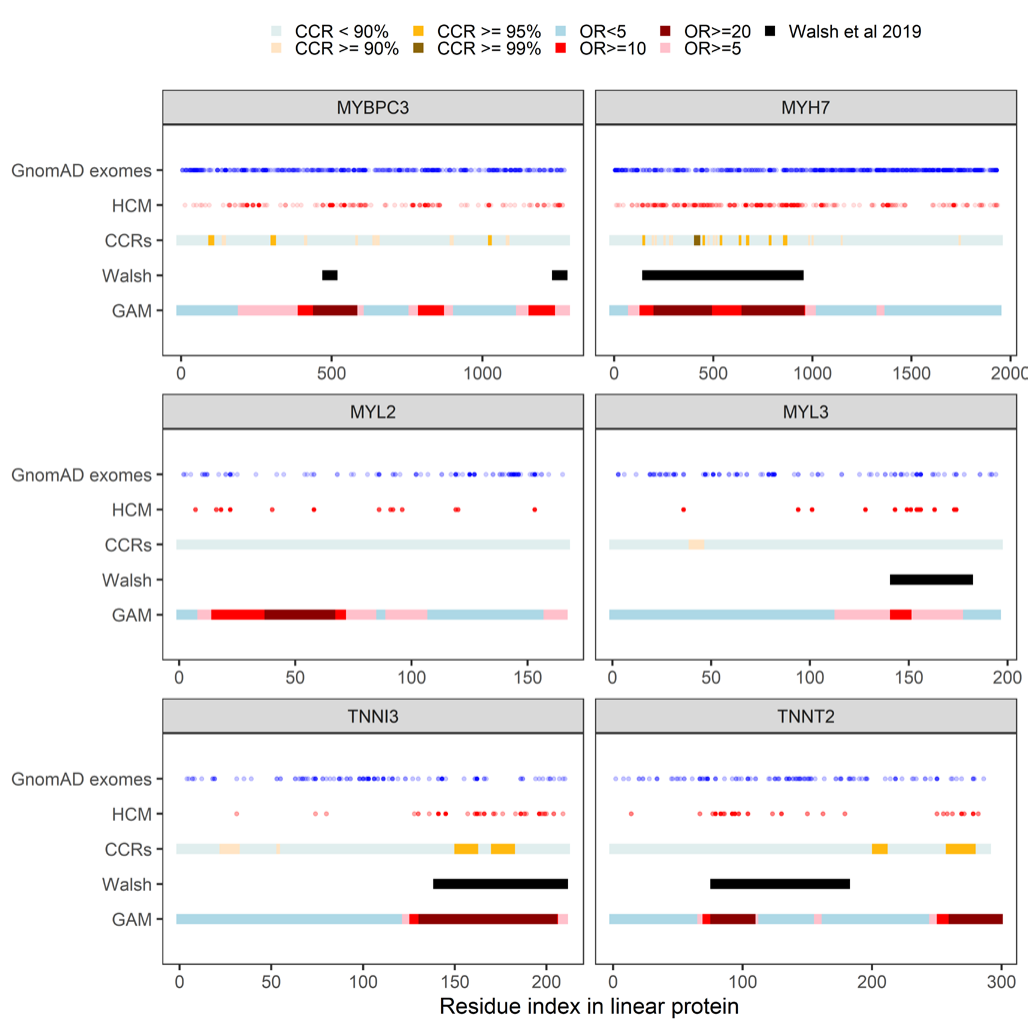
Overlap of GAM hotspots with clusters identified by Walsh *et al* 39, constrained-coding regions identified by Havrilla *et al* 40 and empirical missense variant positions. For six firmly established sarcomeric HCM genes, rare (*popmax* <0.1%) missense variants in GnomAD exomes (blue) and HCM (red) are plotted by their position in the linear protein sequence. GAM predictions from the *hotspot* models are stratified by ORs into moderate (OR ≥20), supporting (OR ≥10) and weak (OR ≥5) evidence clusters. Mutational clusters identified by Walsh *et al* 39 are shown in black. Constrained-coding regions are plotted after stratification by their constraint percentile (≥99%, ≥95%, ≥90% and <90%). GAM, generalised additive model.

Linear predictions from the hotspot models were stratified to delineate moderate (OR ≥20), supporting (OR ≥10) and weak (OR ≥5) regions of pathogenicity potential and compared with 1dC hotspot regions^39^ and CCRs^40^ (figure 5). There was partial cluster overlap for 5/6 genes; however, no cluster was identified by 1dC for *MYL2*, and the *hotspot* model did not identify any clustering in *CSRP3*. Although some overlap with the clusters and CCRs was present in *MYH7* and *TNNI3,* for the most part, there was weak correspondence between this metric and our observed clusters.

Detailed model prediction statistics for the *hotspot* and *hotspot+* model are presented in online supplementary table S5. A web application, *pathogenicity_by_position*, is available to facilitate the exploration of the *hotspot* and *hotspot+* models (R Shiny: https://adamwaring.shinyapps.io/Pathogenicity_by_position). Users can explore alternative models and submit their own missense variants to retrieve predicted ORs and support intervals. A further R package is available for cluster detection and association testing using *BIN-test* and *ClusterBurden* (https://github.com/adamwaring/ClusterBurden). A guide to using *pathogenicity_by_position* is available on the app home page, and instructions for using the R package are available in the documentation and associated vignettes.

10.1136/jmedgenet-2020-106922.supp4 Supplementary data

## Discussion

We present new statistical approaches to incorporate residue position in the analysis of rare missense variants in Mendelian disease genes. Our association tests were well calibrated in simulated data, the *BIN-test* detected significant clustering in almost all firmly established HCM genes, and *ClusterBurden* gave superior power over a simple burden test. Data-driven models were applied to six core sarcomeric genes to estimate mutational hotspots and provides a flexible method for quantitative application of ACMG criteria PM1 and PP3. Our results demonstrate that residue position can be a powerful predictor of both gene and variant pathogenicity. Furthermore, GAMs can quantify the statistical uncertainty surrounding the application of in silico algorithms using gene-specific approaches.

*BIN-test* is a powerful approach to test for variant clustering in known or putative disease genes. *ClusterBurden* is an RVAT with superior power than traditional methods when pathogenic variants cluster in specific protein regions. Both tests keep false-positives below 5% and are rapid to compute, making them scalable for whole-exome scanning of very large datasets like the UK Biobank. Although *ClusterBurden* has slightly reduced power in the absence of clustering, we observed clustering for most well-established HCM genes where missense variants cause disease. Therefore, this method has the potential to be more powerful to detect undiscovered low penetrance genes.

The most significant position signal was observed in the beta myosin heavy chain protein (*MYH7:* ENST00000355349), driven by a substantial excess in the motor domain, a finding that has been long recognised.^42^ High-case and low-control variant density in the carboxy terminus of this protein might lead an observer to hypothesise a regional protective effect on HCM risk (online supplementary figure S3). In sharp contrast, the GAM model predicts a modestly excessive burden (OR ~3) across this entire region discounting this hypothesis (figure 2).

A strong position signal, driven by four potential clusters in domains C1, C3, C7 and C10, was observed in cardiac myosin-binding protein C (figure 2; *MYBPC3:* ENST00000545968). The C1 domain is a suspected myosin S2 and actin-binding site and the C10 domain is a possible *TTN* binding site.^43^ To explore whether the signal was overly driven by high-frequency founder mutations, seven variants with allele counts above 10, p.Arg810His, p.Asp770Asn, p.Glu542Gln, p.Arg502Trp, p.Arg495Gln, p.Glu258Lys and p.Val219Leu (ENST00000545968), were masked in a sensitivity analysis. In their absence, a strong position signal persists (p<3 × 10^−9^) and remaining peak densities overlap with the locations of the (masked) founder mutations (online supplementary figure S4).

Eighty-nine per cent of 27 case variants in cardiac troponin T (TNNT2: ENST00000509001) map to clusters between residues 67–179 and 250–282 (figure 2). The first cluster overlies a previously reported tropomyosin-binding region^44^ and six variants fall between residues 92–110, a region previously noted to impair tropomyosin-dependent functions.^45^ In cardiac troponin I (*TNNI3*: ENST00000344887), 91% of 34 case variants mapped to a cluster spanning residues 128–209. This accords with previous studies documenting carboxy-terminus disease variant clustering.^46^ In myosin light chain 2 (*MYL2*: ENST00000228841), half of 30 case variants cluster between residues 25 and 100, whereas control variants clustered in the C-terminus (figure 2). In myosin light chain 3 (*MYL3*: ENST00000395869), 79% of 14 case variants cluster between residues 125 and 175 (figure 2), whereas control variants were uniformly distributed.

*GAMs* were used to model variant pathogenicity based on mutational hotspots (*hotspot* model) and a combination of mutational hotspots and in silico predictors (*hotspot+* model). GAMs have attractive statistical properties, not necessarily shared by other machine-learning approaches, in that they can produce familiar interpretable results via variant-specific ORs and accompanying 95% CIs. Unlike empirical ORs, based solely on observed frequencies for variants, GAM ORs draw on a much larger pool of information. This permits the estimation of variant-specific ORs whenever the empirical frequencies are uninformative. Furthermore, as the response variable is case status, models are unbiased by previous classifications and account for both penetrance and background rare variation.

Reassuringly, model predictions were positively correlated with expert manually curated classifications. Using a probabilistic approach, we attributed different levels of evidence for the criteria PM1 and PP3. Currently for HCM, criteria PM1 is only applied consistently to *MYH7* as moderate evidence for variants that fall in the residue 181–937 motor domain.^20^ H*otspot* models extend this criteria to five more sarcomeric genes and stratify evidence as supporting (OR ≥10) or moderate (OR ≥20). When in silico predictors were included in the model, evidence was occasionally strong (OR ≥100). This relies on collapsing two ACMG criteria into one, a relevant modification of the current additive guidelines.^16^

Constrained coding regions percentiles^40^ have been calculated across the exome using GnomAD variant data. Intraspecies constraint does not appear to be a definitive metric for the identification of mutational hotspots in HCM. CCRs may be primarily correlated with regions linked to extreme consequences such as embryonic lethality or at least diseases more severe than HCM. Alternatively, the mismatch could be driven by incomplete penetrance, obscuring the constraint in these regions.

Walsh *et al* 39 employed a 1dC to detect local intracohort enrichment of variants with a binomial test. Inconsistent hotspot assignments between 1dC and GAM are likely attributable to small-sample variation, notably for genes with few variants such as *MYL2*. However, there are conceptual differences. The most impactful of these is that 1dC locates clusters using a case-only scan, whereas the *hotspot* models compare cases and controls. Potential consequences of the case-only approach include reduced power to detect multiple clusters as ‘outside-window’ counts are enriched. Furthermore, the GAM approach provides more refined per-residue estimates of pathogenicity, allowing stratification into multiple pathogenicity bands.

In contrast to previous data-driven HCM analyses based on aggregations of clinical reports, adjustment for uneven sequence coverage for the HCM samples was possible due to the availability of BAM files. However, incomplete coverage control is a limitation of this study and more sophisticated adjustment methods may refine hotspot estimation. As a data-driven modelling approach, the GAM estimates become increasingly refined as more data becomes available. Additional improvements to the modelling framework could include the incorporation of historical clustering data as Bayesian priors to further reduce uncertainty in model estimates. Although applied here to HCM as an exemplar proof of concept, ongoing work seeks to extend this method more broadly to Mendelian disease genes with adequate cohort sizes and suitable levels of genetic heterogeneity.

## Conclusions

We present a rare disease/rare variant association test that shows higher theoretical power in synthetic data than traditional burden testing for Mendelian diseases and empirically enhanced power for six sarcomeric HCM genes. We demonstrate how a flexible statistical modelling approach can simultaneously quantify burden and mutational hotspots, with application to firmly established HCM genes. Our approach extends previous studies that defined discrete regions of increased pathogenicity potential to develop a refined map of regional burden across proteins. With the addition of annotation information as covariates in the model, when in silico predictors are used alongside burden and positional information, unique variant-level predictions can outperform published meta-predictors with enhanced sensitivity and specificity.

## Data Availability

Data are available on reasonable request. Due to the confidential nature of some of the research materials supporting this publication, not all of the data can be made accessible to other researchers. Please contact the corresponding author for more information.
