# Supplementary material for: Data-driven modelling of mutational hotspots and in silico predictors in hypertrophic cardiomyopathy
Source: J Med Genet. 2020 Jul 30;58(8):556–64. doi: 10.1136/jmedgenet-2020-106922 (PMC8327322; doi:10.1136/jmedgenet-2020-106922)
Supplement: Supplementary data [file jmedgenet-2020-106922supp001.pdf]

*S1 Table: The ensemble transcript ids for all genes considered in our analyses. For all genes, canonical ensembl transcripts were used.*

| ***Gene*** | ***Transcript*** |
|---|---|
| ACTC1 | ENST00000290378 |
| ACTN2 | ENST00000366578 |
| ANKRD1 | ENST00000371697 |
| BAG3 | ENST00000369085 |
| CRYAB | ENST00000533475 |
| CSRP3 | ENST00000533783 |
| DES | ENST00000373960 |
| DMD | ENST00000357033 |
| DSC2 | ENST00000280904 |
| DSG2 | ENST00000261590 |
| DSP | ENST00000379802 |
| FHL1 | ENST00000394155 |
| FHL2 | ENST00000358129 |
| GLA | ENST00000218516 |
| JUP | ENST00000393931 |
| LAMP2 | ENST00000434600 |
| LMNA | ENST00000368300 |
| MYBPC3 | ENST00000545968 |
| MYH7 | ENST00000355349 |
| MYL2 | ENST00000228841 |
| MYL3 | ENST00000395869 |
| PKP2 | ENST00000070846 |
| PLN | ENST00000357525 |
| PRKAG2 | ENST00000287878 |
| RBM20 | ENST00000369519 |
| SCN5A | ENST00000413689 |
| TAZ | ENST00000299328 |
| TMEM43 | ENST00000306077 |
| TNNC1 | ENST00000232975 |
| TNNI3 | ENST00000344887 |
| TNNT2 | ENST00000509001 |
| TPM1 | ENST00000358278 |
| TTR | ENST00000237014 |
| VCL | ENST00000211998 |

*S2 Table: Implementation details for all statistical tests used in the comparison of type 1 and 2 error.*

| ***Test name*** | ***Implementation (all implemented in R)*** | ***Notes on implementation*** | ***Original paper*** |
|---|---|---|---|
| Fisher's exact test | base::fisher.test | One-sided p-value (*alternative="greater")* | (Fisher 1925) |
| BIN-test | base::chisq.test | Yates' continuity correction | (Pearson 1900) |
| Anderson-Darling | kSamples::ad.test | Asymptotic p-value | (Scholz and Stephens 1987) |
| Kolmorgorov-Smirnov | base::ks.test | P-value approximate for discrete data, Two-sided p-value | (Kolmorgorov 1932) |
| *ClusterBurden* | base::fisher.test + base::chisq.test | Return 0 If either p-value equals 0 | (Fisher 1925) |
| CLUSTER | homepage.ntu.edu.tw/~linwy/CLUSTER.r | PARAMS=[num_perm = 1000, two-sided=TRUE] | (Cheung et al 2012) |
| DoEstRare | DoEstRare::DoEstRare | PARAMS=[alpha=0.05 , c=0.1] | (Persyn et al 2017) |
| WST | assoctesteR::WST | Asymptotic p-value | (Madsen and Browning 2009) |
| C-alpha | assoctesteR::CALPHA | Asymptotic p-value | (Neale et al 2011) |
| SKAT | assoctesteR::SKAT | Asymptotic p-value, PARAMS=[kernel="linear.weighted"] | (Wu et al 2011) |

*S3 Table: A – in-silico predictors considered in this analysis. B – additional features used in each hotspot+ model.*

**A**

| Predictor | Class | Citation |
|---|---|---|
| CADD | Meta | Kircher et al. 2014 |
| Deogen2 | Meta | Raimondi et al. 2017 |
| FATHMM | Meta | Shihab et al. 2013 |
| MetaLR + MetaSVM | Meta | Dong et al. 2015 |
| MPC | Constraint | Samocha et al. 2017 |
| MutationAssessor | Conservation | Reva et al. 2007 |
| MVP | Meta | Hongjian et al. 2018 |
| PhastCons (vertebrate, mammalian, primate) | Conservation | Siepel et al. 2005 |
| PrimateAI | Meta | Sundaram et al. 2018. |
| Provean | Conservation | Choi et al. 2012 |
| REVEL | Meta | Ioannidis et al. 2016. |
| SiPhy 29way | Conservation | Garber et al. 2009 |
| VEST4 | Meta | Carter et al. 2013 |

**B**

| Gene | N predictors | Predictors |
|---|---|---|
| MYH7 | 11 | CADD, Deogen2, MPC, MVP, MetaLR, Provean, PrimateAI, REVEL, FATHMM, PhastCons vertebrate, PhastCons mammalian |
| MYBPC3 | 9 | Deogen2, MVP, MetaLR, MetaSVM, MutationAssessor, REVEL, VEST4, PhastCons primate |
| TNNT2 | 2 | Provean, SiPhy 29way |
| TNNI3 | 1 | MPC score |
| MYL2 | 2 | MutationAssessor, PrimateAI |
| MYL3 | 0 | None |

Adzhubei IA, Schmidt S, Peshkin L, Ramensky VE, Gerasimova A, Bork P, et al. A method and server for predicting damaging missense mutations. Nat Methods. 2010;7(4):248–249. pmid:20354512

Carter H, Douville C, Stenson PD, Cooper DN, Karchin R. Identifying Mendelian disease genes with the variant effect scoring tool. BMC Genomics. 2013;14(Suppl 3):S3.

Choi Y, Sims GE, Murphy S, Miller JR, Chan AP. Predicting the functional effect of amino acid substitutions and indels. PLoS One. 2012;7(10):e46688. pmid:23056405

Daniele Raimondi, Ibrahim Tanyalcin, Julien Ferté, Andrea Gazzo, Gabriele Orlando, Tom Lenaerts, Marianne Rooman, Wim Vranken, DEOGEN2: prediction and interactive visualization of single amino acid variant deleteriousness in human proteins, Nucleic Acids Research, Volume 45, Issue W1, 3 July 2017, Pages W201–W206, https://doi.org/10.1093/nar/gkx390

Dong C, Wei P, Jian X, Gibbs R, Boerwinkle E, Wang K, et al. Comparison and integration of deleteriousness prediction methods for nonsynonymous SNVs in whole exome sequencing studies. Hum Mol Genet. 2015;24(8):2125–2137. pmid:25552646

Garber M, Guttman M, Clamp M, Zody MC, Friedman N, Xie X. Identifying novel constrained elements by exploiting biased substitution patterns. Bioinformatics. 2009;25(12):i54–62.

Hongjian Qi, Chen Chen, Haicang Zhang, John J. Long, Wendy K. Chung, Yongtao Guan, Yufeng Shen. MVP: predicting pathogenicity of missense variants by deep learning. 2018; BioRxiv: doi: https://doi.org/10.1101/259390.

Kaitlin E. Samocha, Jack A. Kosmicki, Konrad J. Karczewski, Anne H. O'Donnell-Luria, Emma Pierce-Hoffman, Daniel G. MacArthur, Benjamin M. Neale, Mark J. Daly. Regional missense constraint improves variant deleteriousness prediction. 2017; BioRxiv: doi: https://doi.org/10.1101/148353.

Kircher M, Witten DM, Jain P, O'Roak BJ, Cooper GM, Shendure J. A general framework for estimating the relative pathogenicity of human genetic variants. Nat Genet. 2014;46(3):310–315. pmid:24487276

Nilah M. Ioannidis, Joseph H. Rothstein, Vikas Pejaver, *et al.*. REVEL: An Ensemble Method for Predicting the Pathogenicity of Rare Missense Variants. The American Journal of Human Genetics, Volume 99, Issue 4, 2016, Pages 877-885.

Reva B, Antipin Y, Sander C. Determinants of protein function revealed by combinatorial entropy optimization. Genome Biol. 2007;8(11):R232. pmid:17976239

Shihab HA, Gough J, Cooper DN, Stenson PD, Barker GL, Edwards KJ, et al. Predicting the functional, molecular, and phenotypic consequences of amino acid substitutions using hidden Markov models. Hum Mutat. 2013;34(1):57–65. pmid:23033316

Siepel A, Bejerano G, Pedersen JS, Hinrichs AS, Hou M, Rosenbloom K, Clawson H, Spieth J, Hillier LW, Richards S, et al. Evolutionarily conserved elements in vertebrate, insect, worm, and yeast genomes. Genome Res. 2005;15(8):1034–50.

Sundaram, L., Gao, H., Padigepati, S.R. et al. Predicting the clinical impact of human mutation with deep neural networks. Nat Genet 50, 1161–1170 (2018). https://doi.org/10.1038/s41588-018-0167-z

*S4 Table: Type 1 error and power assessed via simulated data for proposed methods and published alternatives.*

| | *Clustering model:* | **Uniform** | | **One cluster** | | **Multiple clusters** | |
|---|---|---|---|---|---|---|---|
| | *Protein length:* | **500** | **1000** | **500** | **1000** | **500** | **1000** |
| **Cases** | **Unique variants** | 13.1 (5.8) | 26.1 (11.6) | 9.7 (6.2) | 19.4 (12.6) | 12.4 (6.3) | 24.8 (12.7) |
| **Controls** | **Unique variants** | 81.9 (82.2) | 163.9 (164.5) | 85.7 (85.9) | 171.1 (172.2) | 86.0 (86.3) | 172.3 (172.9) |
| | **Burden odds-ratio** | 2.49 (1.02) | 2.49 (1.03) | 1.71 (1.02) | 1.71 (1.03) | 2.28 (1.01) | 2.26 (1.02) |
| | | | | | | | |
| **Burden** | **Fisher-exact test** | 0.8 (0.042) | 0.97 (0.05) | 0.39 (0.044) | 0.58 (0.05) | 0.69 (0.042) | 0.87 (0.053) |
| **Two-sample goodness-of-fit tests** | ***BIN-test*** | 0.058 (0.051) | 0.058 (0.047) | 0.31 (0.051) | 0.52 (0.05) | 0.45 (0.05) | 0.73 (0.047) |
| | **Kolmorgorov-Smirnov** | 0.036 (0.023) | 0.039 (0.03) | 0.16 (0.024) | 0.31 (0.032) | 0.26 (0.026) | 0.49 (0.033) |
| | **Anderson-Darling** | 0.056 (0.051) | 0.054 (0.049) | 0.18 (0.05) | 0.31 (0.05) | 0.27 (0.05) | 0.48 (0.049) |
| **Position-informed RVATs** | ***ClusterBurden*** | 0.72 (0.05) | 0.94 (0.05) | 0.48 (0.049) | 0.7 (0.053) | 0.76 (0.047) | 0.93 (0.052) |
| | **DoEstRare** | 0.8 (0.058) | 0.96 (0.062) | 0.46 (0.06) | 0.64 (0.06) | 0.74 (0.058) | 0.9 (0.063) |
| | **CLUSTER** | 0.82 (0.054) | 0.97 (0.056) | 0.42 (0.055) | 0.61 (0.059) | 0.71 (0.053) | 0.88 (0.057) |
| **Standard RVATs** | **C-alpha** | 0.75 (0.051) | 0.94 (0.056) | 0.42 (0.053) | 0.59 (0.058) | 0.7 (0.051) | 0.87 (0.057) |
| | **WST** | *NA* (0.17) | *NA* (0.089) | *NA* (0.16) | *NA* (0.086) | *NA* (0.16) | *NA* (0.08) |
| | **SKAT** | *NA* (0.18) | *NA* (0.2) | *NA* (0.18) | *NA* (0.21) | *NA* (0.18) | *NA* (0.2) |
