# Supplementary material for: Data-driven modelling of mutational hotspots and in silico predictors in hypertrophic cardiomyopathy
Source: J Med Genet. 2020 Jul 30;58(8):556–64. doi: 10.1136/jmedgenet-2020-106922 (PMC8327322; doi:10.1136/jmedgenet-2020-106922)

S1 Figure: A) Hotspot model predictions and B) Hotspot+ model predictions (ORS) stratified by OMGL classifications with 95% confidence intervals.

Model predictions from both the hotspot-model (burden and clustering) and the hotspot+ model (burden, clustering and functional prediction scores) are stratified by pathogenicity classifications made by Oxford Medical Genetics laboratory. Only rare-missense variants (popmax < 0.1%) are plotted excluding most likely benign and benign variants. Spearman rank correlation coefficients are presented to indicate the strength of correlation between the numerical predicted ORs and the ordinal ACMG classes. 95% confidence intervals are displayed alongside point estimates and the x-axis is presented on a log scale.

A

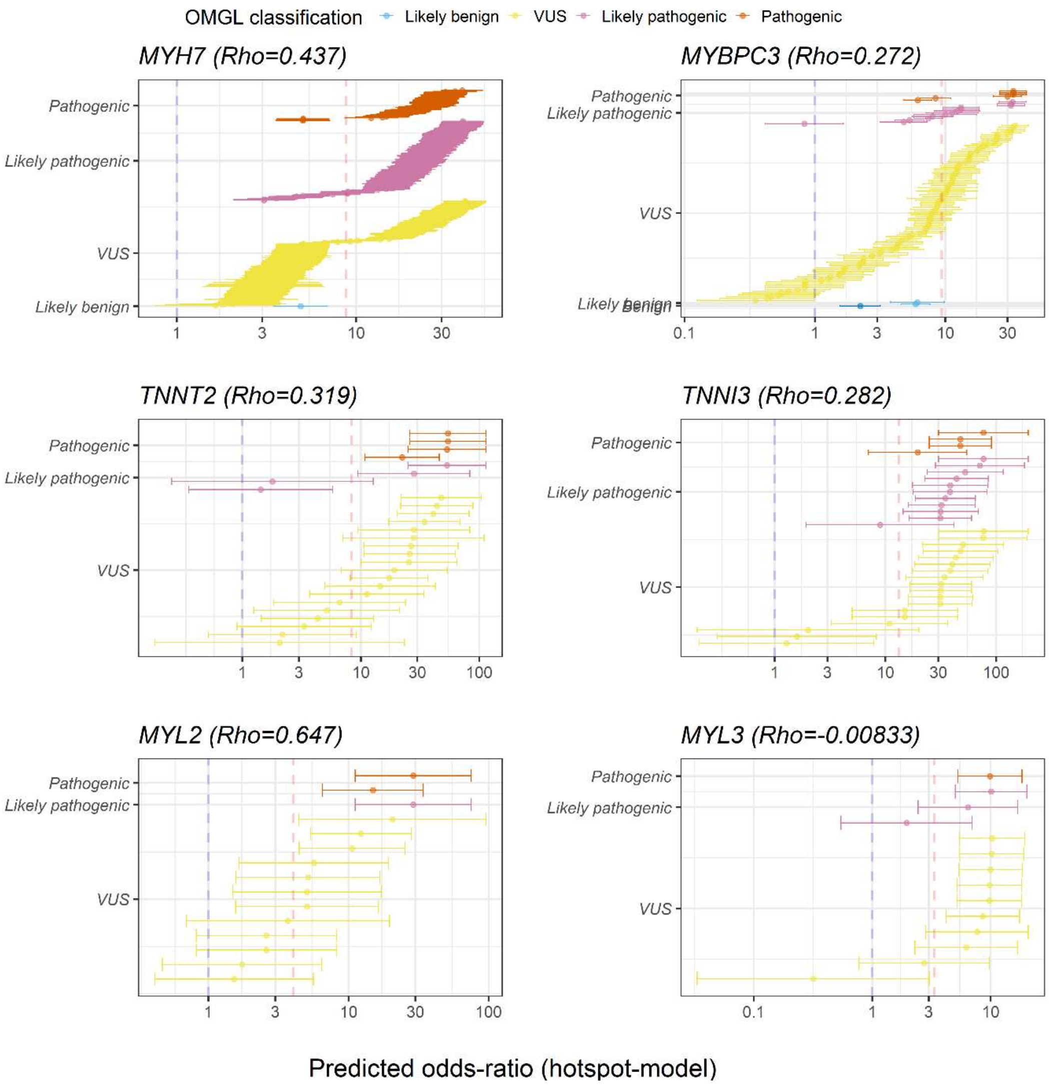

B

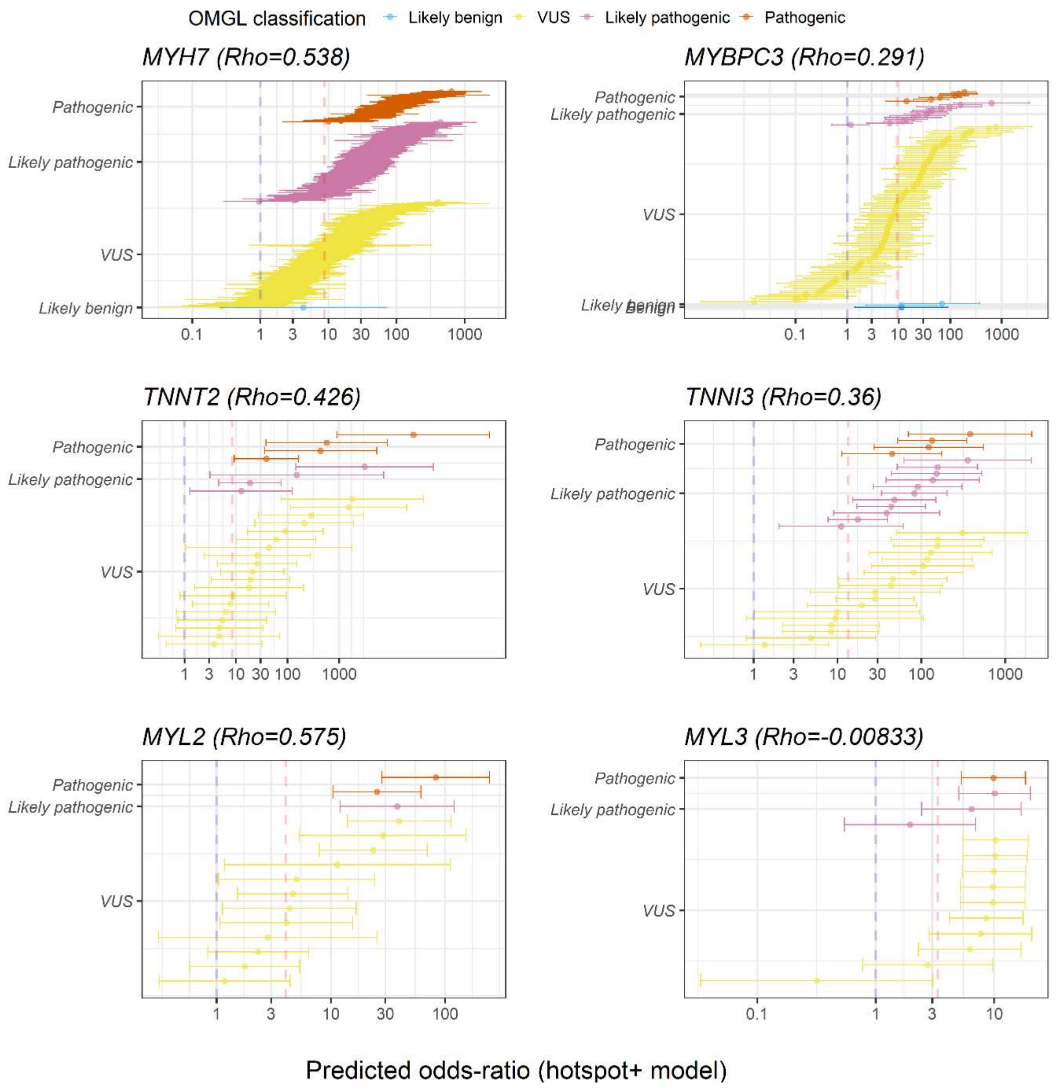
OMGL classification
Likely benign
VUS
Likely pathogenic
Pathogenic
MYH7 (Rho=0.538)
MYBPC3 (Rho=0.291)
TNNT2 (Rho=0.426)
TNNI3 (Rho=0.36)
MYL2 (Rho=0.575)
MYL3 (Rho=-0.00833)
Predicted odds-ratio (hotspot+ model)

S2 Figure: Full mean and standard deviation AUC table for all models considered.

For each gene, the hotspot and hotspot+ model are compared to each individual *in-silico* predictor from dbNSFP. Each model is trained on the same HCM-GnomAD case-control missense variants filtered at a GnomAD population maximum frequency of 0.01%.

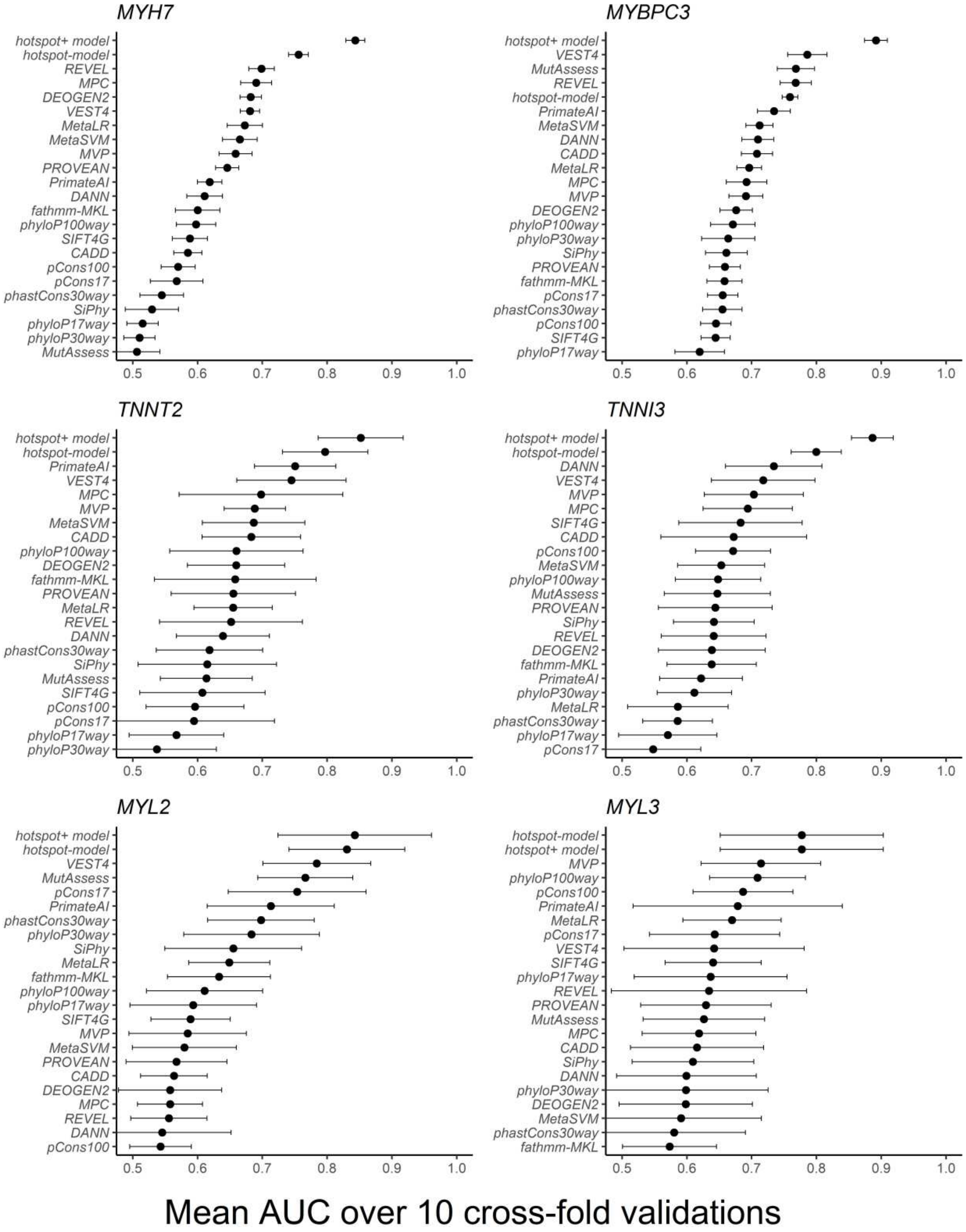

S3 Figure: Distribution and risk predictions for rare-missense *MYH7* variants in our case-control cohort.

The variant positions and training data for the GAM are a case cohort of 5,338 hypertrophic cardiomyopathy cases and 125,748 gnomAD controls. The density plot (B) may give the impression that there is an excess of control variants in the C-terminus of the *MYH7* protein; however the GAM model (A) resolves this potential misinterpretation and clearly shows an odds-ratio greater than 1 for the entire protein.

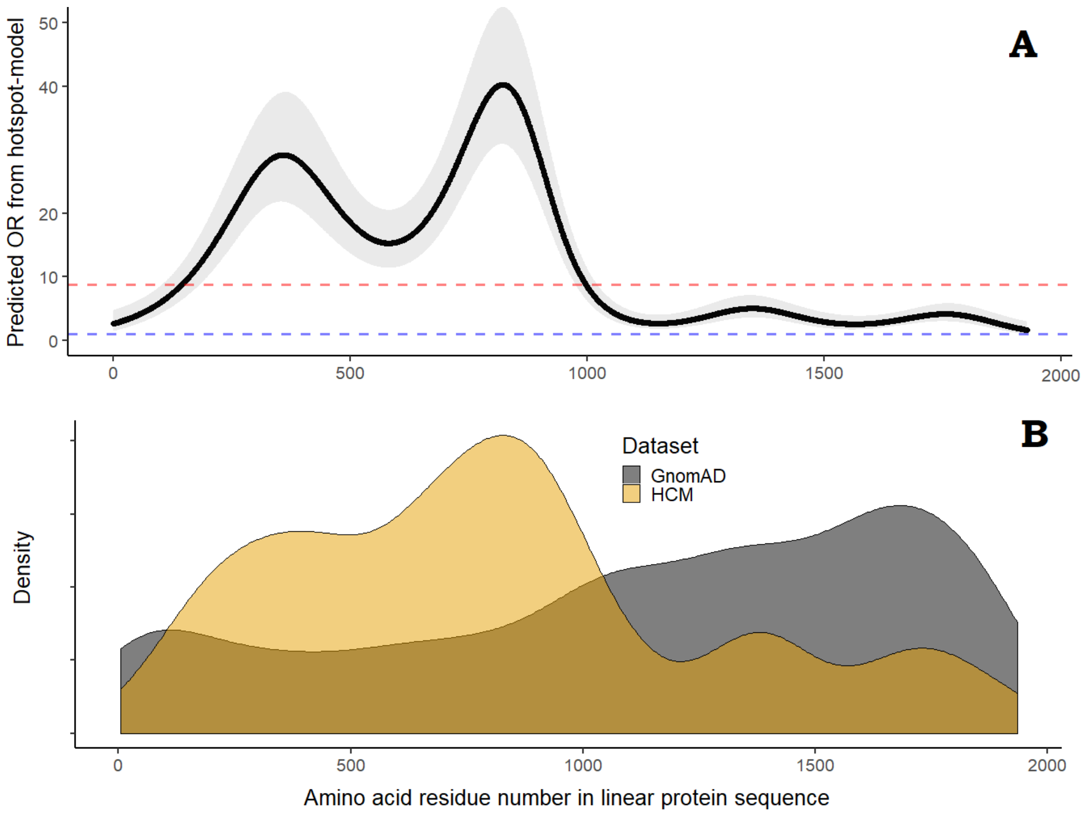

S4 Figure: Rare-missense variant clustering in *MYBPC3* with and without potential founder mutations.

Variant clustering model (*hotspot-model*) are generated for three different frequency filtering strategies. The model identifies four discrete regions with high pathogenic potential regardless of whether the founder mutations are included in the analysis. However, the magnitude of the predicted ORs are higher under normal filtering conditions (e.g. *popmax* < 0.01%).

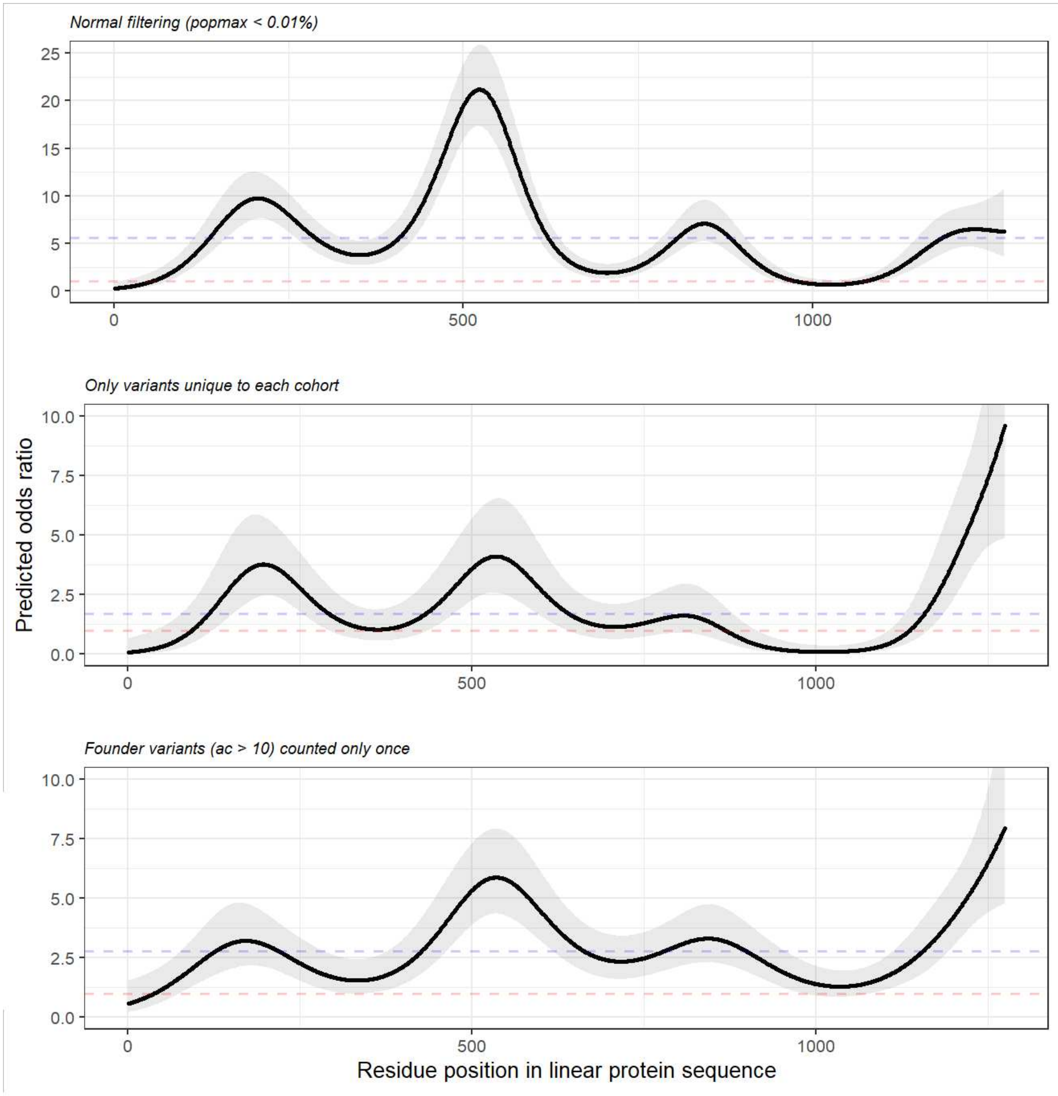

Supplement: Supplementary data [file jmedgenet-2020-106922supp003.pdf]
